# Social isolation as a significant risk factor for depression in colorectal cancer patients post-colostomy: a cross-sectional study

**DOI:** 10.3389/fpsyt.2025.1588314

**Published:** 2025-05-20

**Authors:** Jianmei Zou, Haina Jia, Jing Wang, Yanxia Li

**Affiliations:** ^1^ Department of Oncology, Zhongshan People’s Hospital, Zhongshan, China; ^2^ Department of General Surgery, Nanfang Hospital, Southern Medical University, Guangzhou, China; ^3^ Department of Gastrointestinal Tumor Surgery, Guangzhou Medical University Cancer Hospital, Guangzhou, China; ^4^ Department of Cardiothoracic Surgery, Zhuhai People’s Hospital, Zuhai, China

**Keywords:** colorectal cancer, colostomy, social isolation, depression, cross-sectional study

## Abstract

**Objective:**

Colorectal cancer is a leading global health concern, with significant physical and psychosocial impacts on patients. Many CRC patients undergo colostomy procedures, which can lead to lifestyle changes and an increased risk of depression. Social isolation, a detachment from social networks, has been linked to depression in various chronic illnesses but remains underexplored in this specific patient group. This study aims to investigate the association between social isolation and depressive symptoms in patients with colorectal cancer following colostomy.

**Methods:**

A cross-sectional study design was employed, enrolling colorectal cancer patients who underwent colostomy from January 2020 to January 2023. Clinical and questionnaire data were collected. The Chinese version of the Patient Health Questionnaire (PHQ-9) was utilized to assess depression, while the Lubben Social Network Scale (LSNS-6) evaluated social isolation. Demographic characteristics, clinical variables, psychosocial adaptation, and social support were also gathered. Univariate and multivariate logistic regression analyses, along with subgroup analyses, were conducted to identify risk factors influencing depression.

**Results:**

A total of 290 patients were included, among which 139 were diagnosed with depression. Both univariate and multivariate analyses indicated that tumor stages III-IV (OR=1.94, 95% CI: 1.14-3.30) and prolonged colostomy duration (OR=4.04, 95% CI: 1.87-8.72) are independent risk factors for depression, while social isolation (OR=0.24, 95% CI: 0.13-0.44) is a significant protective factor. The prevalence of depression was significantly higher in the social isolation group compared to the non-social isolation group (58.8% *vs*. 43.8%, P=0.024). The prevalence of depression was significantly higher in the social isolation group compared to the non-social isolation group (58.8% *vs*. 43.8%, P=0.024). Subgroup analyses revealed significant interactions, with social isolation showing stronger inverse associations with depression in males, individuals aged <60 or >70, divorced/widowed/single patients, and those with low social support.

**Conclusion:**

Social isolation represents a crucial risk factor for depression in colorectal cancer patients following colostomy. Healthcare professionals should prioritize the assessment of patients’ social isolation and implement targeted interventions to mitigate the risk of depression.

## 1 Introduction

Colorectal cancer (CRC) ranks as the third most prevalent malignancy globally, with over 1.9 million new diagnoses reported in 2020, accounting for 9.4% of all cancer-related fatalities (1, 2). Advancements in surgical techniques have led to approximately 30%-50% of CRC patients requiring either permanent or temporary ostomy procedures to sustain physiological functions (3, 4). It is noteworthy that different ostomy types may present distinct psychosocial challenges. And adequate social support mitigates the negative psychological impact of stressful life events by influencing cognitive appraisal and coping processes (5).

While these surgical interventions significantly enhance survival rates, patients face the challenge of long-term ostomy bag usage, which necessitates substantial lifestyle modifications. The unpredictability of bowel movements not only disrupts daily activities but also predisposes individuals to complications such as peristomal skin issues (6). The resultant physiological changes, body image disturbances, and self-care burdens frequently contribute to serious psychosocial adjustment challenges (7). Research indicates that the prevalence of depression among patients following ostomy surgery ranges from 38%-52%, notably higher than in the general cancer population (8). This depressive state not only diminishes quality of life but can also adversely affect treatment adherence, thereby increasing the risk of postoperative complications and ultimately leading to reduced survival rates (9). Consequently, identifying the core risk factors for depression in ostomy patients and devising targeted intervention strategies has emerged as a critical focus within cancer rehabilitation.

Social isolation is characterized by an individual’s detachment from social networks, resulting in insufficient communication and interaction with others. This phenomenon encompasses multiple dimensions, including social and emotional isolation, and has been significantly correlated with an elevated risk of depression among chronic illness patients (10). From a theoretical perspective, social support theory suggests that strong social connections provide emotional and instrumental resources that buffer against psychological distress (11). Similarly, stress and coping theory posits that social isolation may impair an individual’s ability to effectively manage illness-related stressors, thereby increasing vulnerability to depression.

For patients with colorectal cancer who have undergone ostomy procedures, alterations in body image, the stigma associated with their condition, and lifestyle changes may lead to either voluntary or involuntary reductions in social interactions, thereby fostering a state of social isolation (12). Previous studies have identified social isolation as an independent predictor of depression in patients with breast and prostate cancers (13). Additionally, existing literature has established associations between social isolation and various adverse health outcomes, including cardiovascular disease, cognitive decline, and increased all-cause mortality (14).

Current investigations into the factors influencing depression among ostomy patients predominantly emphasize demographic characteristics (such as age and gender) and clinical variables (such as tumor stage and type of ostomy). However, the role of social isolation as a potentially significant influencing factor remains underexplored. This study aims to systematically examine the relationship between social isolation and depressive symptoms in patients post-ostomy following colorectal cancer surgery through a cross-sectional design.

## 2 Methods

### 2.1 Setting

This investigation employed a cross-sectional design, conducted from January 2020 to January 2023.

The study employed a consecutive sampling method, enrolling patients who underwent ostomy procedures for colorectal cancer at the ostomy outpatient clinic and during postoperative follow-up. Clinical data and questionnaire responses were collected. The study received approval from the ethics committee of our institution, and informed consent was obtained from all participants and their families (15). However, we acknowledge that it cannot establish causal relationships due to the simultaneous assessment of exposure and outcome.

### 2.2 Study subjects

**Inclusion Criteria**

1. Patients diagnosed with colorectal cancer through pathological examination and who have undergone ostomy surgery. Pathological diagnosis serves as the gold standard for confirming colorectal cancer, ensuring the accuracy of disease identification among participants.
2. Patients aged 18 years or older, capable of independent thought and expression, ensuring their ability to accurately comprehend and respond to relevant questions.
3. Patients with clear consciousness and normal communication abilities, enabling effective participation in the study.

**Exclusion Criteria**

1. Patients with a history of other severe mental disorders, such as schizophrenia or bipolar disorder, to prevent interference from additional psychiatric conditions in the assessment of depression.
2. Patients exhibiting severe cognitive impairments that hinder their ability to accurately understand questionnaire content or participate in the study.
3. Patients who have experienced significant stressors within the past three months, such as major familial changes or economic crises, to eliminate potential confounding factors affecting the relationship between social isolation and depression.

### 2.3 Sample size calculation

Sample size was determined using PASS 2021 software based on logistic regression analysis based on Pourhoseingholi (16). Assuming an expected odds ratio (OR) for social isolation of 2.0 (α=0.05, power 90%, two-sided test), with a depression prevalence of 40% in the control group (patients without social isolation) and an exposure rate (social isolation) of 30%, the calculated minimum sample size was 248 cases. Accounting for a 15% non-response or invalid data rate, the final target sample size was established at 300 cases.

### 2.4 Outcome and exposure indicators

Depression served as the primary outcome measure, assessed using the Chinese version of the Patient Health Questionnaire (PHQ-9) (17), which has been validated for reliability and validity in the Chinese cancer population. The total score ranges from 0 to 27, with a score of ≥10 indicating clinically significant depression. Its Cronbach’s α coefficient is 0.86, and the retest reliability is 0.82.

Social isolation was the primary exposure variable, evaluated using the Lubben Social Network Scale (LSNS-6) (18), which consists of two components: family and friend networks, totaling six items. Total scores range from 0 to 30, with higher scores reflecting better social network quality. A total score of ≤12 indicates social isolation. The LSNS-6 has a Cronbach’s α coefficient of 0.83, and the retest reliability is 0.84.

### 2.5 Covariates

#### 2.5.1 Demographic Characteristics

Age, gender, education level (categorized as ≤ junior high, high school, ≥ college), marital status, and family income (grouped into tertiles).

#### 2.5.2 Clinical Variables

Tumor stage (AJCC 8th Edition stages I–IV), type of ostomy (permanent/temporary), and postoperative complications (Clavien-Dindo grade ≥ II).

#### 2.5.3 Social Psychological Adjustment

Social psychological adjustment was evaluated using the Chinese version of the Ostomate Adjustment Inventory (OAI-20) (19), encompassing three dimensions: positive emotions, negative emotions, and social life adjustment, with a total of 20 items. Each item is rated on a scale from 0 to 4, resulting in a total score ranging from 0 to 80; higher scores indicate better social psychological adjustment. Scores below 40 indicate low adjustment, 40–59 indicate moderate adjustment, and ≥60 indicate high adjustment. Its Cronbach’s α coefficient is 0.85, and the retest reliability is 0.89.

#### 2.5.4 Social Support

Social support was assessed using the Medical Outcomes Study Social Support Survey (MOS-SSS) (20). The MOS-SSS comprises four dimensions and 20 items, including tangible support, informational and emotional support, social interaction cooperation, and emotional support. Each item is rated on a 5-point Likert scale, with a maximum total score of 100; higher scores indicate greater perceived levels of social support. A total score of ≥60 indicates high social support, while scores below this threshold indicate low social support. Its Cronbach’s α coefficient is 0.87, and the retest reliability is 0.85.

### 2.6 Quality control

Standardized training was conducted for the medical personnel or research assistants involved in the survey. Training content included the study’s objectives, questionnaire details, survey methodology, communication techniques, and essential precautions. Through simulated surveys and practical exercises, investigators were ensured proficiency in the survey methods, enabling them to collect data accurately and objectively.

Surveys were conducted during the 12-month postoperative follow-up, either in hospital wards or outpatient settings. Investigators provided a detailed explanation of the study’s purpose and significance to patients, obtained their consent, and distributed the questionnaires while offering guidance for completion. For patients with visual impairments or low educational levels facing difficulties in completing the questionnaire, investigators filled out the questionnaire according to the patients’ responses, ensuring comprehension of the questions while promoting independent responses. Upon completion, questionnaires were checked on-site for completeness and accuracy, with any omissions or ambiguities promptly addressed. The PHQ-9 typically toke 2–10 minutes to complete. The LSNS-6 takes approximately 3–10 minutes to complete. The OAI toke approximately 3–10 minutes to complete. The MOS-SSS usually toke 5–10 minutes for completion.

### 2.7 Statistical methods

Descriptive statistics were employed to summarize patients’ demographic information, social isolation scores, and depression scores. For the 10 excluded invalid questionnaires, listwise deletion was applied as they lacked complete baseline data. For the remaining included participants, missing item-level data in questionnaires (e.g., single missing items in PHQ-9) were handled by prorating when at least 80% of items were completed; otherwise, the questionnaire was excluded from analysis. Continuous variables were expressed as mean ± standard deviation, while categorical variables were reported as frequencies and percentages to elucidate the basic characteristics and variable distribution among study subjects. Chi-square tests were utilized to analyze the relationship between categorical variables (such as gender, education level, marital status, etc.) and depression. Independent sample t-tests or ANOVA were conducted to compare depression score differences across various groups (e.g., different levels of social isolation) to identify factors potentially associated with depression. Factors demonstrating statistical significance in univariate analyses were incorporated into a multivariate logistic regression model, with depression as the dependent variable and other factors as independent variables. This analysis aimed to ascertain whether social isolation constitutes an independent risk factor for depression in ostomy patients following colorectal cancer surgery, calculating odds ratios (OR) and 95% confidence intervals (CI) to evaluate the strength of the association between factors and depression. Significance was determined at P < 0.05 for all analyses.

## 3 Results

### 3.1 Demographic and clinical data

A total of 300 patients were enrolled, with 10 invalid questionnaires, resulting in 290 participants included in the final analysis: 139 patients with depression and 151 patients without depression. Comparisons of demographic, clinical, and psychological data revealed that the postoperative duration was significantly longer in patients with depression than in those without, the proportion of patients in stages I-II was significantly lower among depressed patients, and the rates of social isolation and low MOS-SSS levels were significantly higher in depressed patients (all p-values < 0.05). Demographic, clinical, and psychological data for depressed and non-depressed patients are summarized in **Table 1**.

**Table 1:** Analysis of demographic, clinical, and psychological data of patients with depression.

Characteristic	PHQ-9		Values	
	<12, N = 151^1^	≥12, N = 139^1^	Statistic	p-value
**Age**	62 ± 8	62 ± 7	0.02	0.982^2^
**Age group**			0.17	0.919^3^
60 to 70	60 (39.7%)	57 (41.0%)		
less than 60	69 (45.7%)	64 (46.0%)		
more than 70	22 (14.6%)	18 (12.9%)		
**Gender**			0.00	0.991^3^
Female	64 (42.4%)	59 (42.4%)		
Male	87 (57.6%)	80 (57.6%)		
**Marital status**			1.85	0.174^3^
Divorced/Widowed/Unmarried	31 (20.5%)	38 (27.3%)		
Married	120 (79.5%)	101 (72.7%)		
**Income**			0.06	0.969^3^
<5000	49 (32.5%)	46 (33.1%)		
>10000	39 (25.8%)	37 (26.6%)		
5000-10000	63 (41.7%)	56 (40.3%)		
**Duration**			19.64	<0.001^3^
12 to 24	75 (49.7%)	75 (54.0%)		
less than 12	52 (34.4%)	20 (14.4%)		
more than 24	24 (15.9%)	44 (31.7%)		
**Tumor stage**			9.50	0.002^3^
I-II	70 (46.4%)	40 (28.8%)		
III-IV	81 (53.6%)	99 (71.2%)		
**Stoma type**			1.87	0.172^3^
Permanent	108 (71.5%)	89 (64.0%)		
Temporary	43 (28.5%)	50 (36.0%)		
**Complication**			0.21	0.646^3^
No	130 (86.1%)	117 (84.2%)		
Yes	21 (13.9%)	22 (15.8%)		
**OAI-20**			2.39	0.122^3^
High	95 (62.9%)	75 (54.0%)		
Low	56 (37.1%)	64 (46.0%)		
**MOS-SSS**			28.37	<0.001^3^
High	120 (79.5%)	69 (49.6%)		
Low	31 (20.5%)	70 (50.4%)		
**Social isolation**			5.18	0.023^3^
No	118 (78.1%)	92 (66.2%)		
Yes	33 (21.9%)	47 (33.8%)		

^1^Mean ± SD; n (%).

^2^Welch Two Sample t-test.

^3^Pearson’s Chi-squared test.

### 3.2 Comparison of baseline characteristics between social isolation and non-social isolation groups

Following the assessment, participants were classified into two groups: the social isolation group (LSNS-6 ≥ 12) and the non-social isolation group (LSNS-6 < 12). Comparative analyses of baseline characteristics revealed that the proportions of individuals with high income, those classified as stages I-II, and those with temporary ostomies were significantly lower in the social isolation group compared to the non-social isolation group. Additionally, the postoperative duration was significantly longer in the social isolation group, while the rates of low MOS-SSS scores and depression were significantly higher (all p-values < 0.05). The demographic, clinical, and psychological data for patients in both groups are summarized in **Table 2**.

**Table 2:** Demographic, clinical, and psychological data of patients with social isolation and non-social isolation.

Characteristic	LSNS-6			
	<12, N = 210^1^	≥12, N = 80^1^	Statistic	p-value
**Age**	62 ± 7	63 ± 8	-0.57	0.567^2^
**Age group**			4.28	0.118^3^
60 to 70	90 (42.9%)	27 (33.8%)		
less than 60	96 (45.7%)	37 (46.3%)		
more than 70	24 (11.4%)	16 (20.0%)		
**Gender**			0.67	0.415^3^
Female	86 (41.0%)	37 (46.3%)		
Male	124 (59.0%)	43 (53.8%)		
**Marital status**			0.09	0.766^3^
Divorced/Widowed/Unmarried	49 (23.3%)	20 (25.0%)		
Married	161 (76.7%)	60 (75.0%)		
**income**			35.49	<0.001^3^
<5000	48 (22.9%)	47 (58.8%)		
>10000	67 (31.9%)	9 (11.3%)		
5000-10000	95 (45.2%)	24 (30.0%)		
**Duration**			10.07	0.007^3^
12 to 24	105 (50.0%)	45 (56.3%)		
less than 12	62 (29.5%)	10 (12.5%)		
more than 24	43 (20.5%)	25 (31.3%)		
**Tumor stage**			6.40	0.011^3^
I-II	89 (42.4%)	21 (26.3%)		
III-IV	121 (57.6%)	59 (73.8%)		
**Stoma type**			7.39	0.007^3^
Permanent	133 (63.3%)	64 (80.0%)		
Temporary	77 (36.7%)	16 (20.0%)		
**Complication**			0.18	0.674^3^
No	180 (85.7%)	67 (83.8%)		
Yes	30 (14.3%)	13 (16.3%)		
**OAI-20**			1.08	0.299^3^
high	127 (60.5%)	43 (53.8%)		
low	83 (39.5%)	37 (46.3%)		
**MOS-SSS**			64.57	<0.001^3^
high	166 (79.0%)	23 (28.8%)		
low	44 (21.0%)	57 (71.3%)		
**Depression**			5.18	0.023^3^
No	118 (56.2%)	33 (41.3%)		
Yes	92 (43.8%)	47 (58.8%)		

^1^Mean ± SD; n (%).

^2^Welch Two Sample t-test.

^3^Pearson’s Chi-squared test.

### 3.3 Analysis of risk factors for depression

**Figure 1:**
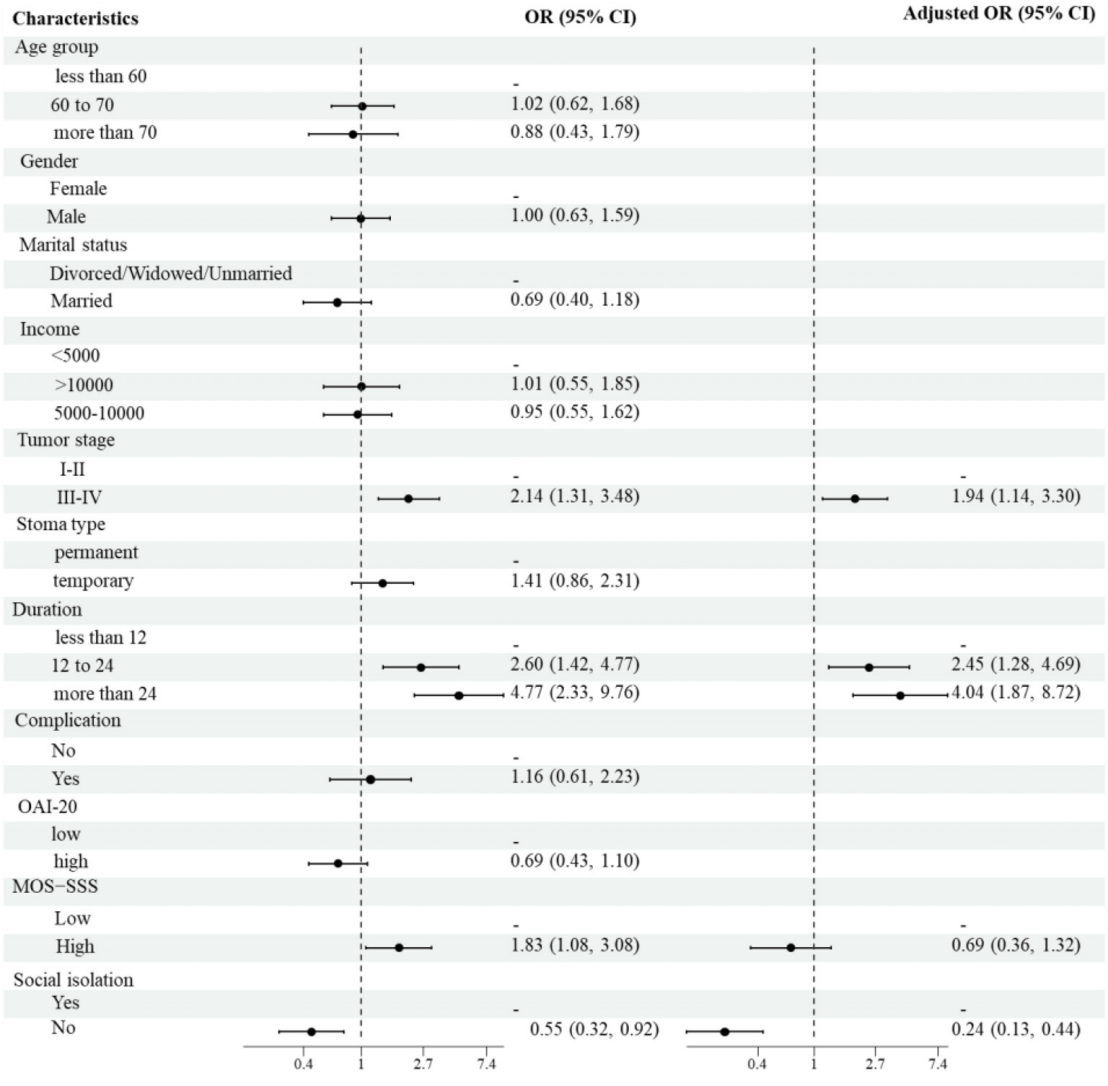
Forest plot of risk factors for depression.

Univariate and multivariate logistic regression analyses were performed to identify the risk factors associated with depression. The univariate analysis indicated that tumor stages III-IV, prolonged ostomy duration, low social support levels, and social isolation were significant risk factors for depression. The multivariate analysis identified tumor stages III-IV (OR=1.94, 95% CI: 1.14-3.30, P=0.014), prolonged ostomy duration (OR=4.04, 95% CI: 1.87-8.72, P<0.001), and social isolation (OR=0.24, 95% CI: 0.13-0.44, P<0.001) as independent risk factors for depression. The results of both the univariate and multivariate analyses are presented in **Table 3** and illustrated in **Figure 1**.

**Table 3:** Univariate and multivariate logistic regression analysis of depression.

Characteristic	Univariable			Multivariable		
	OR^1^	95% CI^1^	p-value	OR^1^	95% CI^1^	p-value
Age group						
less than 60	—	—				
60 to 70	1.02	0.62, 1.68	0.925			
more than 70	0.88	0.43, 1.79	0.729			
Gender						
Female	—	—				
Male	1.00	0.63, 1.59	0.991			
Marital status						
Divorced/Widowed/Unmarried	—	—				
Married	0.69	0.40, 1.18	0.175			
Income						
<5000	—	—				
>10000	1.01	0.55, 1.85	0.973			
5000-10000	0.95	0.55, 1.62	0.843			
Tumor stage						
I-II	—	—		—	—	
III-IV	2.14	1.31, 3.48	0.002	1.94	1.14, 3.30	0.014
Stoma type						
permanent	—	—				
temporary	1.41	0.86, 2.31	0.173			
Duration						
less than 12	—	—		—	—	
12 to 24	2.60	1.42, 4.77	0.002	2.45	1.28, 4.69	0.007
more than 24	4.77	2.33, 9.76	<0.001	4.04	1.87, 8.72	<0.001
Complication						
No	—	—				
Yes	1.16	0.61, 2.23	0.646			
OAI-20						
low	—	—				
high	0.69	0.43, 1.10	0.122			
MOS-SSS						
low	—	—		—	—	
high	1.83	1.08, 3.08	0.024	0.69	0.36, 1.32	0.261
Social isolation						
Yes	—	—		—	—	
No	0.55	0.32, 0.92	<0.001	0.24	0.13, 0.44	<0.001

^1^OR, Odds Ratio; CI, Confidence Interval.

### 3.4 Subgroup analysis of the impact of social isolation on depression

In the overall population analysis, the odds ratio (OR) for the association between social isolation and depression was 0.55 (95% CI: 0.32-0.92, P=0.024), indicating that patients without social isolation (LSNS-6 ≥12) had lower odds of depression compared to socially isolated patients. The interaction effect by age was significant (P for interaction = 0.034), with individuals under 60 years (OR = 0.39, P = 0.018) and those over 70 years (OR = 0.19, P = 0.017) showing lower odds of depression. The interaction effect by gender approached significance (P for interaction = 0.053), revealing a stronger association among males (OR = 0.34, P = 0.004). The interaction effect based on marital status also approached significance (P for interaction = 0.071), indicating a more significant association in individuals who were divorced, widowed, or never married (OR = 0.20, P = 0.011). Interaction effects related to income, tumor stage, type of ostomy, duration, complications, and OAI-20 were not significant (P for interaction > 0.05). However, the interaction effect with the MOS-SSS was significant (P for interaction = 0.003), suggesting that the association between social isolation and depression was stronger among individuals with low social support (OR = 4.40, P = 0.02), where social isolation was associated with higher depression risk. The findings from the subgroup analysis investigating the impact of social isolation on depression are detailed in **Table 4**.

**Table 4:** Subgroup analysis of the impact of social isolation on depression.

Subgroup	No^1^	Yes^1^	Crude OR (95% CI)	P value	P for interaction
Overall	92/210 (43.8)	47/80 (58.8)	1.83 (1.08-3.08)	0.024	
Age group					0.034
60 to 70	45/90 (50.0)	12/27 (44.4)	0.80 (0.34-1.90)	0.613	
less than 60	40/96 (41.7)	24/37 (64.9)	2.58 (1.18-5.68)	0.018	
more than 70	7/24 (29.2)	11/16 (68.8)	5.34 (1.35-21.14)	0.017	
Gender					0.053
Female	41/86 (47.7)	18/37 (48.6)	1.04 (0.48-2.25)	0.921	
Male	51/124 (41.1)	29/43 (67.4)	2.96 (1.43-6.16)	0.004	
Marital status					0.071
Divorced/Widowed/Unmarried	22/49 (44.9)	16/20 (80.0)	4.91 (1.43-16.83)	0.011	
Married	70/161 (43.5)	31/60 (51.7)	1.39 (0.77-2.52)	0.278	
Income					0.441
<5000	17/48 (35.4)	29/47 (61.7)	2.94 (1.28-6.77)	0.011	
>10000	32/67 (47.8)	5/9 (55.6)	1.37 (0.34-5.54)	0.661	
5000-10000	43/95 (45.3)	13/24 (54.2)	1.43 (0.58-3.51)	0.436	
Tumor stage					0.437
I-II	29/89 (32.6)	11/21 (52.4)	2.28 (0.87-5.97)	0.095	
III-IV	63/121 (52.1)	36/59 (61.0)	1.44 (0.77-2.71)	0.258	
Stoma type					0.422
permanent	54/133 (40.6)	35/64 (54.7)	1.77 (0.97-3.22)	0.064	
temporary	38/77 (49.4)	12/16 (75.0)	3.08 (0.91-10.39)	0.07	
Duration					0.145
12 to 24	50/105 (47.6)	25/45 (55.6)	1.37 (0.68-2.77)	0.374	
less than 12	14/62 (22.6)	6/10 (60.0)	5.14 (1.27-20.82)	0.022	
more than 24	28/43 (65.1)	16/25 (64.0)	0.95 (0.34-2.67)	0.926	
Complication					0.996
No	78/180 (43.3)	39/67 (58.2)	1.82 (1.03-3.21)	0.039	
Yes	14/30 (46.7)	8/13 (61.5)	1.83 (0.48-6.90)	0.373	
OAI-20					0.74
high	52/127 (40.9)	23/43 (53.5)	1.66 (0.83-3.33)	0.154	
low	40/83 (48.2)	24/37 (64.9)	1.98 (0.89-4.42)	0.093	
MOS-SSS					0.003
high	66/166 (39.8)	3/23 (13.0)	0.23 (0.06-0.80)	0.02	
low	26/44 (59.1)	44/57 (77.2)	2.34 (0.99-5.55)	0.053	

^1^no. of events/total no. (%).

## 4 Discussion

Patients with ostomies following colorectal cancer surgery are at an elevated risk of depression due to physiological changes, body image disturbances, and social isolation. This study systematically investigated the association between social isolation and depression in ostomy patients using a cross-sectional design, highlighting its role as an independent risk factor and further exploring subgroup heterogeneity.

The results demonstrate a significant correlation between social isolation (LSNS-6 ≤ 12) and depression among ostomy patients (OR = 0.55, 95% CI: 0.32-0.92). In the multivariate model, this risk remained significant even when adjusted for tumor stage (III-IV, OR = 1.94) and postoperative duration (>24 months, OR = 4.04). According to social support theory, robust social networks provide emotional, informational, and practical assistance, aiding individuals in coping with stress and adverse emotions (21, 22). Among ostomy patients, 68% reported reducing social interactions due to concerns about ostomy bag leakage or odor, which heightened their sensitivity to negative evaluations and exacerbated social withdrawal, resulting in social isolation (23). Qualitative research indicates that some patients, fearing ostomy-related issues in social contexts, opt for isolation, leading to diminished external engagement (24). Such self-isolation diminishes their social circles, further entrenching them in a state of social withdrawal. Patients experiencing social isolation often lack support from family, friends, and the broader community, making them more vulnerable to feelings of helplessness and despair, ultimately increasing their risk of depression. Additionally, social isolation may adversely affect lifestyle and health behaviors (9, 25), as individuals who are socially isolated tend to engage in less physical activity, experience poorer sleep quality, and adopt unhealthy dietary habits (26). These lifestyle alterations can further compromise physical health, potentially influencing neuroendocrine and immune function and heightening susceptibility to depression.

Our subgroup analyses revealed an unexpected pattern: social isolation was associated with lower odds of depression in younger (<60 years) and older (>70 years) age groups, males, and divorced/widowed/single individuals. This counterintuitive finding warrants careful consideration. One potential explanation is a “survivor effect,” where socially isolated individuals in these subgroups may have developed unique coping mechanisms or resilience over time, mitigating the typical negative impact of isolation (22). Alternatively, the LSNS-6 may not fully capture the qualitative aspects of social interactions, such as participation in ostomy-specific support groups, which could provide emotional support despite quantitative measures indicating isolation (12). Additionally, unmeasured variables, such as personality traits (e.g., introversion) or prior mental health history, might mediate these relationships (27). Future research should explore these dynamics using mixed-methods designs to disentangle the complex interplay between isolation and depression in specific populations.

It is important to note that while our study identifies social isolation as an independent risk factor for depression, the cross-sectional design limits our ability to establish a definitive causal relationship. To address the question of whether depression was caused by social isolation or locally advanced disease, we conducted multivariate analyses that controlled for tumor stage and other clinical variables. These analyses allowed us to isolate the effect of social isolation on depression, even in the presence of advanced disease. However, the complex interplay between social isolation and advanced disease cannot be fully disentangled in a cross-sectional study. Future research with longitudinal designs or interventional studies that modify social isolation while controlling for disease stage could provide further insights into the causal pathways.

Prior research on factors influencing depression in ostomy patients has primarily focused on demographic and clinical variables, such as age, gender, tumor stage, and ostomy type (28). This study is the first to comprehensively examine social isolation, addressing a notable gap in psychosocial research within this domain. Studies in other chronic illness populations have established a strong link between social isolation and increased depression risk (9, 27, 29, 30). However, this investigation specifically validates the impact of social isolation on depression in the distinct cohort of ostomy patients following colorectal cancer surgery, revealing differences across subgroups by age, gender, marital status, and social support levels, thereby enriching the existing literature.

Furthermore, this study identified tumor stages III-IV and prolonged ostomy duration as independent risk factors for depression. Previous research consistently demonstrates that patients with advanced tumors face higher mortality risks and more complex treatment pathways, which correlate with disease progression and poorer prognoses, thereby increasing depression risk (31, 32). Prolonged ostomy care and lifestyle adjustments contribute to sustained psychological stress for both patients and caregivers, exacerbating depressive symptoms (33). Regarding ostomy type, previous studies have yielded inconsistent findings, with some suggesting higher depression rates in patients with permanent ostomies (34, 35). In contrast, this study found no significant association between ostomy type and depression, potentially due to differences in sample selection and ostomy care support across studies. While few studies have explicitly examined social isolation in ostomy patients using the LSNS-6, broader research on loneliness and social support aligns with our findings. For instance, Gu et al. (36) reported that perceived social support mediated the relationship between isolation and depression in breast cancer patients, suggesting similar mechanisms may apply to ostomy populations. Our study extends this literature by quantifying the isolated-depression association and identifying high-risk subgroups, such as those with low social support or advanced disease. The link between social isolation and depression in ostomy patients likely involves multifaceted psychological and physiological pathways. Psychologically, isolation may exacerbate maladaptive cognitive styles, such as rumination or negative self-evaluation, which are known to amplify depressive symptoms (26). Physiologically, chronic social isolation has been linked to dysregulation of the hypothalamic-pituitary-adrenal (HPA) axis and elevated inflammatory markers (e.g., IL-6, CRP), which are implicated in depression. For ostomy patients, the added stress of body image concerns and stigma may further activate these pathways, creating a vicious cycle (23). Interventions targeting both social connectivity and stress reduction (e.g., mindfulness-based therapies) could thus be particularly beneficial.

The findings of this study carry important clinical implications. Healthcare professionals should routinely utilize the LSNS-6 to assess patients’ social isolation status, enabling timely identification of those at high risk for early intervention (23). For individuals experiencing social isolation, establishing peer support groups for ostomy patients can facilitate communication and mutual support, allowing patients to share experiences and encourage one another, thus improving social connectivity (37). Providing professional psychological counseling can assist patients in navigating changes in body image and feelings of stigma, enhancing psychological resilience and reducing depression risk. Additionally, patients with advanced tumor stages and prolonged ostomy durations should receive increased attention and support, including comprehensive education on disease management and ostomy care to help them adapt to the lifestyle changes associated with their conditions.

This study has several strengths, including a robust sample size calculated *a priori*, validated instruments (PHQ-9, LSNS-6), and comprehensive adjustment for clinical and psychosocial confounders. The use of subgroup analyses to explore heterogeneity adds nuance to our understanding of social isolation’s impact. However, the cross-sectional design limits causal inference, and future longitudinal studies are needed to confirm these associations.

This study is not without limitations. The cross-sectional design precludes the establishment of a causal relationship between social isolation and depression; future research may benefit from prospective cohort studies or interventional designs to further validate these findings. The sample was drawn from specific ostomy outpatient clinics and postoperative follow-up departments, potentially introducing selection bias; subsequent studies should broaden sample sources to enhance the generalizability of the results. Additionally, the specific neurobiological mechanisms through which social isolation influences depression remain to be fully elucidated, warranting future investigations that integrate neuroscientific methodologies to provide a more robust theoretical foundation for intervention strategies.

## 5 Conclusion

This study underscores social isolation as a significant and modifiable risk factor for depression in colorectal cancer patients following colostomy. The findings highlight that patients with advanced tumor stages, prolonged ostomy duration, and limited social networks are particularly vulnerable. Importantly, our results call for routine screening for social isolation in clinical practice using validated tools like the LSNS-6, enabling early identification of at-risk individuals. Targeted interventions—such as structured peer support programs, psychosocial counseling, and enhanced social support initiatives—should be prioritized, especially for high-risk subgroups (e.g., males, older adults, and those with low baseline support).

While this study provides critical insights, longitudinal and interventional research is needed to confirm causality and evaluate the effectiveness of isolation-mitigation strategies. By integrating psychosocial care into standard ostomy management, healthcare providers can significantly improve mental health outcomes and overall quality of life for these patients.

## Data Availability

The original contributions presented in the study are included in the article/supplementary material. Further inquiries can be directed to the corresponding author.
